# Supercritical CO_2_ Foaming of Poly(3-hydroxybutyrate-*co*-4-hydroxybutyrate)

**DOI:** 10.3390/polym14102018

**Published:** 2022-05-15

**Authors:** Tao Zhang, Yunjae Jang, Eunhye Lee, Sooan Shin, Ho-Jong Kang

**Affiliations:** 1 Department of Polymer Science and Engineering, Dankook University, 152 Jukjeon-ro, Suji-gu, Yongin-si 16890, Gyeonggi-do, Korea; taozhang1214@gmail.com; 2 CJ Cheiljedang Corporation, 55 Gwanggyo-ro, 42 beon-gil, Yeongtong-gu, Suwon-si 16495, Gyeonggi-do, Korea; yunjae.jang@cj.net (Y.J.); eunhye.lee@cj.net (E.L.); sooanshin@gmail.com (S.S.)

**Keywords:** poly(3-hydroxybutyrate-*co*-4-hydroxybutyrate), supercritical carbon dioxide, P(3HB-*co*-4HB) foams, expansion ratio, chain extender

## Abstract

The supercritical carbon dioxide foaming characteristics of the biodegradable polymer poly(3-hydroxybutyrate-co-4-hydroxybutyrate) (P(3HB-*co*-4HB)) are studied for environmentally friendly packaging materials. The effect of the 4HB composition of the P(3HB-*co*-4HB) copolymers on the foaming conditions such as pressure and temperature is studied and the density and the expansion ratio of the resulting P(3HB-*co*-4HB) foam are together evaluated. The increase in the 4HB content reduces the crystallinity and tan δ value of P(3HB-*co*-4HB) required for the growth of the foam cells. Therefore, the foaming temperature needs to be lower to retain a suitable tan δ value of P(3HB-*co*-4HB) for foaming. It was found that P(3HB-*co*-4HB) with less crystallinity showed better formability and cell uniformity. However, foaming is not possible regardless of the foaming temperature when the 4HB content of P(3HB-*co*-4HB) is over 50%, due to the high tan δ value. A lower foam density and higher expansion ratio can be obtained with crystalline P(3HB-*co*-4HB) of low 4HB content, compared with non-crystalline P(3HB-*co*-4HB) of high 4HB content. The expansion ratio of P(3HB-*co*-4HB) foams can be increased slightly by using a chain extender, due to the lowing of crystallinity and tan δ. This is most effective in the case of P(3HB-*co*-4HB), whose 4HB content is 16%.

## 1. Introduction

Polyhydroxyalkanoates (PHAs) are biodegradable polymers produced by microorganisms and have very wide applicability as an alternative material for synthetic polymers, which cause environmental pollution as they are degraded under diverse earth environments, contrary to other biodegradable polymers [1,2]. Due to their excellent biocompatibility and in vivo degradation properties, their application is being extended to medical materials [3]. The developed PHA products are melt processed films [4,5] or foams [6] for environmentally friendly packing material and research is being carried out regarding their application to medical materials, such as biodegradable sutures from electrospinning [7] and scaffolds for tissue engineering from 3D printing [8], etc.

Poly(3-hydroxybutyrate) (P3HB) was the first biodegradable polymer to be studied among the PHAs, but had the limitation of having a narrow window for melt processing, as thermal degradation occurs very easily [9]. Furthermore, its crystallization rate is very low, resulting in large spherulites with the interface among these resulting in brittleness and the crystallization continually occurs during solidification, making the stable production of molded products difficult [10]. To overcome these problems, blending with biodegradable polymers that are compatible with P3HB, such as polylactic acid (PLA) and polybutylene succinate (PBS) [11,12], modification of the structure by branching or crosslinking through the addition of diverse additives [13,14], and the bacterial synthesis of copolymers from biodegradable monomers [15] have been suggested. The comonomers widely used with 3-hydroxybutyrate in the study of PHA copolymers are 3-hydroxyvalerate to prepare poly(3-hydroxybutyrate-*co*-3-hydroxyvalerate) [16], 4-hydroxybutyrate to prepare poly(3-hydroxybutyrate-*co*-4-hydroxybutyrate) [17], and hydroxyhexanoate to prepare poly(3-hydroxybutyrate-*co*-hydroxyhexanoate) [18].

Due to the higher price of PHAs compared with commodity polymers, they have been considered for use in high priced medical materials, but the decrease in production costs through mass production is resulting in higher possibilities of extending its application to packaging material [19,20]. As a relatively smaller amount of PHA is needed to prepare foamed material with a porous structure, the development of packaging material from PHA using foaming agents is being carried out [21]. Polymer foams can be made by the addition of diverse chemical foaming agents [22,23], using environmentally friendly supercritical fluid in melt extrusion [24,25], and the introduction of supercritical fluid to beads in the solid state to prepare foam beads [26]. Obtained foam beads can be molded by chest injection molding [27]. There are two typical processes to produce bead foam, continuous process and batch process using foam extrusion and autoclave, respectively. In the autoclave bead, polymer beads are saturated with a physical blowing agent, and pressure in the autoclave is suddenly released to expand the beads. Bead foaming is an appropriate processing method for semi-crystalline polymers, such as polypropylene (PP) [28], polyurethane (TPU) [29], and PLA [30]. The use of carbon dioxide supercritical fluid to maintain the environmentally friendly nature of biodegradable polymers is reported mostly in PLA foams [31,32,33,34,35], and in the case of PHA, the melt extrusion foaming of PHBV with supercritical CO_2_ is being carried out [36,37]. However, research on the foaming of P3HB has not been reported due to the lack of foamability. It is necessary to develop an eco-friendly supercritical CO_2_ process using biodegradable P(3HB-*co*-4HB) with improved chain flexibility.

The effect of 4HB content in the bead foam process using supercritical CO_2_ on the processing conditions and the resulting structure of the foams is studied. The effect of foaming temperature and pressure on the cell structure is studied in detail by evaluating the foam density, expansion ratio, etc. to explore the possibilities of application of these foams to packaging material. The effect of the addition of chain extending agents on the foam structure is also studied.

## 2. Experimental

**Figure 1:**
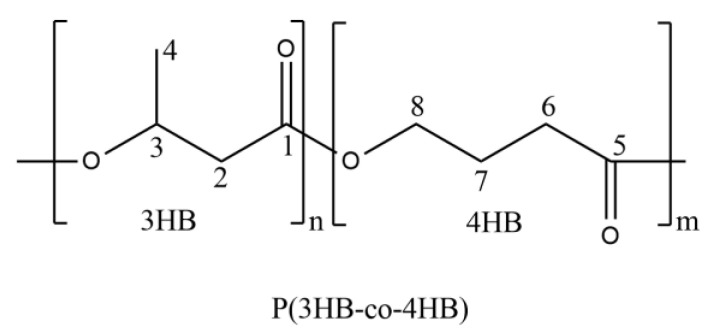
Chemical structure of P(3HB-*co*-4HB).

The P(3HB-*co*-4HB) copolymer used in this study is a biodegradable polymer produced by CJ Cheiljedang, among which those with a 4HB content of 10–16% are confirmed to be crystalline and those with a 4HB content of 30 or 53.7% are confirmed to be non-crystalline. The chemical structure and 4HB content, along with molecular weights and heat of melting, are shown in Figure 1 and Table 1, respectively. A multifunctional styrene-acrylic oligomer (Joncryl, ADR 4370) purchased from BASF was used as a chain extender for the required control of the viscoelasticity of P(3HB-*co*-4HB) in the supercritical CO_2_ foaming process.

P(3HB-*co*-4HB) of different 4HB contents were melt processed in an internal mixer (Haake, Rheomix600, Verden, Germany) at 20 rpm for 10 min at 140 °C, minimizing the thermal degradation to prepare samples of the same thermal history. When a 5 phr chain extender was added, the melt processing was carried out at 180 °C, which is the temperature at which the chain extender can react. The obtained P(3HB-*co*-4HB) samples were compression molded in a compression molding machine (QMESYS, QM900, Anyang, Korea) by heating at 140 °C for 2 min, raising the pressure to 8 MPa, and molded for 2 min, then quenched in water to obtain 1T thick 150 mm × 150 mm sheets. The sheets were cut into 5 mm × 5 mm pieces to use as beads for foaming.

The tan δ of P(3HB-*co*-4HB) was evaluated by measuring 1 mm × 2.5 mm sheets on a dynamic mechanical thermal analyzer (TA, DMA Q800/2980, New Castle, DE, USA) in the 30–140 °C range, at the oscillation frequency of 1 MHz.

**Figure 2:**
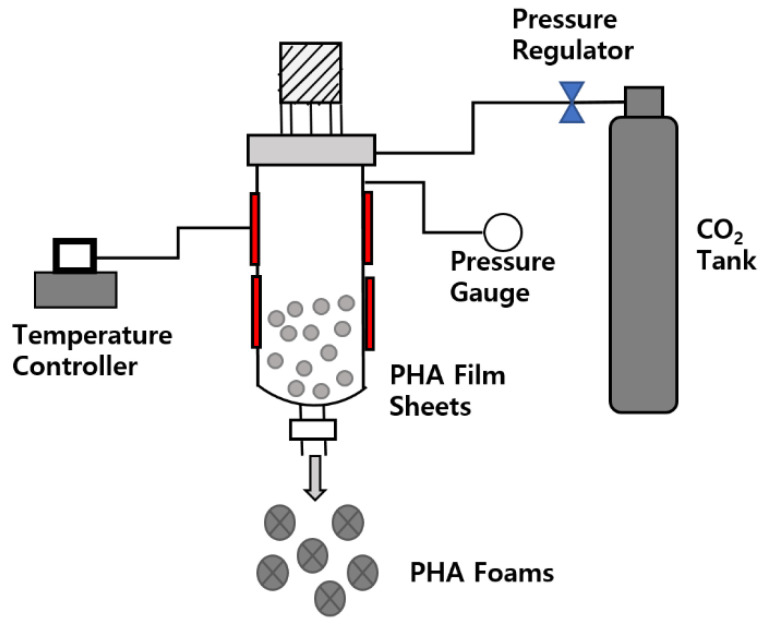
Schematic of supercritical CO_2_ assisted foaming process.

The P(3HB-*co*-4HB) foam beads were prepared by adding 10 g of beads to a lab-made autoclave, as shown in Figure 2, diffusing supercritical CO_2_ into P(3HB-*co*-4HB) for 60 min at the supercritical condition of CO_2_ of 50–135 °C and 70–100 bar, then releasing the pressure abruptly to atmospheric pressure.

The structure of the P(3HB-*co*-4HB) foams was evaluated with SEM pictures, taken on a scanning electron microscope (Hitachi, SEM S-5200, Hitachi, Tokyo, Japan) and by measuring the density before foaming ($\rho_{p}$) and the density after foaming ($\rho_{f}$) from which the expansion ratio (Φ) was calculated according to the following Equation (1).

(1) $$\Phi =\frac{\rho_{p}}{\rho_{f}}$$

Cell density $\left(N_{f}\right)$ refers to the number of cells (n) per cubic centimeter (A) in a foamed sample and can be defined by the following Equation (2).

(2) $$\;\;\;\;\;\;\;\;\;N_{f}={\left(\frac{n}{A}\right)}^{\frac{3}{2}}\;X\;\Phi \;\;\;\;\;\;\;$$

To measure the resilience rate of the crystalline P(3HB-*co*-10% 4HB) foam beads and noncrystalline P(3HB-*co*-30% 4HB) foam beads, the thickness of the foam beads was first determined as $T_{0}$, then after compressing the foam beads to 50% its thickness and releasing the pressure after 30 min, the thickness was measured as T; the resilience rate was calculated according to the following Equation (3).

(3) $$\;\;\;\;\;\;\;\;\;R=\frac{T}{T_{0}}\;\times \;100\;\left(\%\right)\;\;\;$$

## 3. Results and Discussion

### 3.1. Foamability of P(3HB-co-4HB) Copolymer

**Figure 3:**
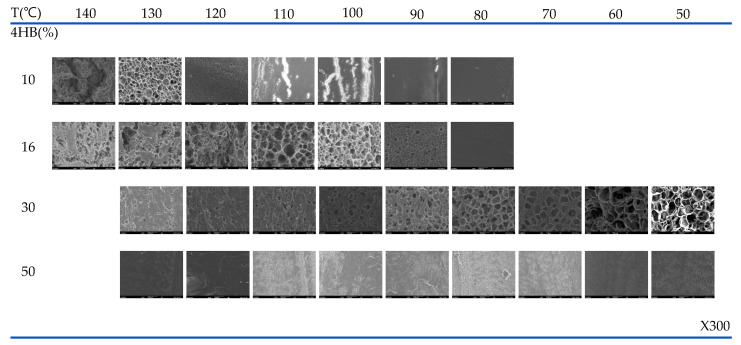

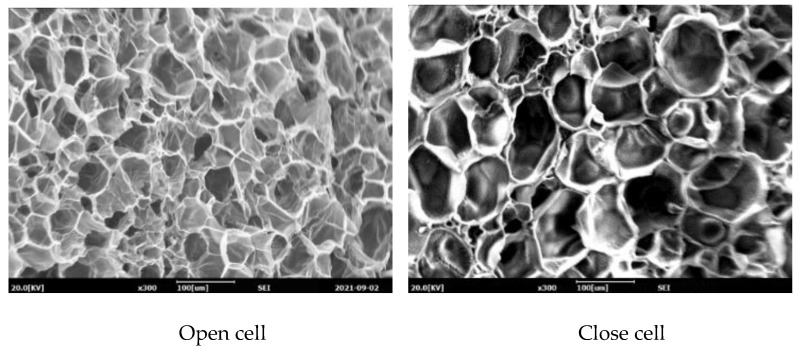
SEM micrographs of P(3HB-*co*-4HB) with different 4HB content foamed at 90 bar.

The SEM micrographs that show the possibilities of cell formation in the foams prepared in the range 50–130 °C at 90 bar for different 4HB contents are shown in Figure 3. As can be observed, the temperature at which the cells develop depends on the 4HB content. In the case of P(3HB-*co*-10% 4HB), whose relative crystallinity is highest, (23.97 J/g) as shown in Table 1, cell structure develops when processed at 130 °C, while in the case of P(3HB-*co*-16% 4HB), whose relatively crystallinity is lower (3.99 J/g), cell structure develops when processed at 100–110 °C. P(3HB-*co*-30% 4HB) is non-crystalline and the cell structure develops at a lower foaming temperature of 50–80 °C, but cell structure does not develop in the case of P(3HB-*co*-50% 4HB) in the range studied.

In supercritical foaming, the supercritical fluid diffuses into the polymer to create nuclei and they grow to form cells [38]. Nucleation and cell growth are affected by the crystalline/non-crystalline structure of the polymer and viscoelasticity, specifically, reasonable crystallinity and tan δ value under the foaming conditions are required for nucleation and continuous growth of the cell. In crystalline P(3HB-*co*-4HB), poor solubility and diffusivity of CO_2_ in crystallites caused the lowering of nucleation of the cell and prohibits cell growth, which resulted in less formability.

**Figure 4:**
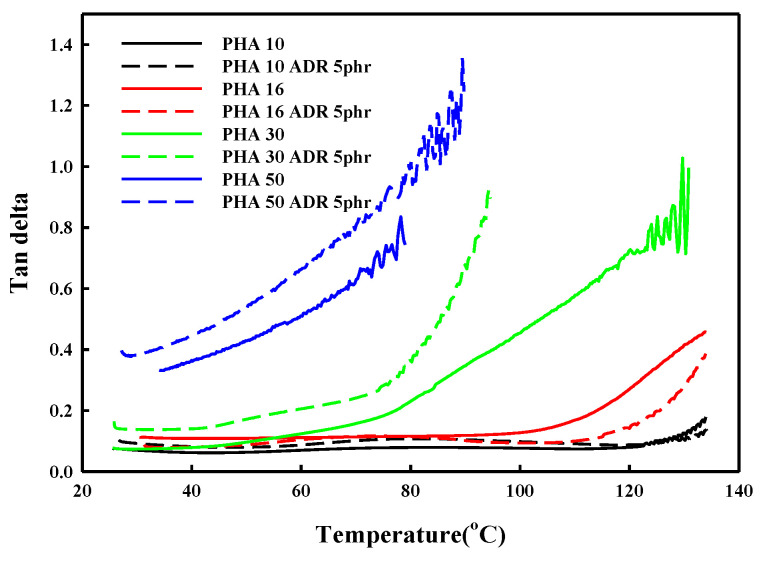
DMA thermograms of various P(3HB-*co*-4HB) with and without ADR.

The change in tan δ with temperature for the P(3HB-*co*-4HB) is shown in Figure 4. The tan δ value and crystallinity are closely related to the chain mobility in the solid state. An increase in the tan δ value is observed with an increase in temperature and 4HB content. The tan δ is the ratio of loss modulus to storage modulus and shows a drastic change at various transition temperatures. The drastic change in the tan δ with temperature can be observed as the temperature reaches the melting transition temperature of crystalline P(3HB-*co*-4HB) or the rubber transition temperature of non-crystalline P(3HB-*co*-4HB). The drastic change occurs at around 130 °C and 110 °C for crystalline P(3HB-*co*-10% 4HB) and P(3HB-*co*-16% 4HB), respectively, as their melting temperature depends on the 4HB content. On the other hand, the change can be observed above 50 °C and 30 °C for non-crystalline P(3HB-*co*-30% 4HB) and P(3HB-*co*-50% 4HB), respectively, where the mobility of the non-crystalline chains start to increase. The tan δ value increases with 4HB content in P(3HB-*co*-4HB) and the change in the tan δ value also increases at the respective transition temperatures. In the case of the P(3HB-*co*-4HB) copolymers, the chain mobility of 4HB is greater than that of 3HB, resulting in a higher tan δ value and a more sensitive change with temperature. P(3HB-*co*-50% 4HB) exhibits a higher tan δ value than other P(3HB-*co*-4HB) copolymers regardless of the temperature, due to the too much chain flexibility of the 4HB chain. The adequate tan δ value required for cell growth in supercritical fluid foaming can be indirectly assessed by the tan δ value reflecting the polymer’s viscoelasticity. As observed in Figure 3, for copolymers with a 4HB content up to 30%, the tan δ values below the transition temperature where the chains show a drastic change in their mobility are very similar, with the values being around 0.1. Therefore, foaming is expected to be possible below the transition where chain mobility is increased, which is 130 °C for P(3HB-*co*-10% 4HB), 110 °C for P(3HB-*co*-16% 4HB), and below 80 °C for P(3HB-*co*-30% 4HB). However, P(3HB-*co*-50% 4HB) exhibits a tan δ value above 0.3, regardless of the temperature reflecting the extremely chain mobility; thus, cell growth in the foaming process is not possible. As observed in Figure 3, in the case of P(3HB-*co*-10% 4HB) of low 4HB content, the temperature range where foaming is possible is very narrow around 130 °C, as the stiffness of the chains is high, resulting in a drastic decrease in the mobility of chains. In the case of P(3HB-*co*-16% 4HB) where the flexibility of the chains is increased with an increase in 4HB content, foaming is possible in a relatively broader temperature range of 100–110 °C, and P(3HB-*co*-30% 4HB), which has the highest chain mobility, has the widest window where foaming is possible of 50–90 °C. Therefore, it is evident that in non-crystalline P(3HB-*co*-4HB) an adequate tan δ related to chain mobility above the glass transition temperature is an important factor in foaming.

### 3.2. Characteristics of P(3HB-co-4HB) Foams

**Figure 5:**
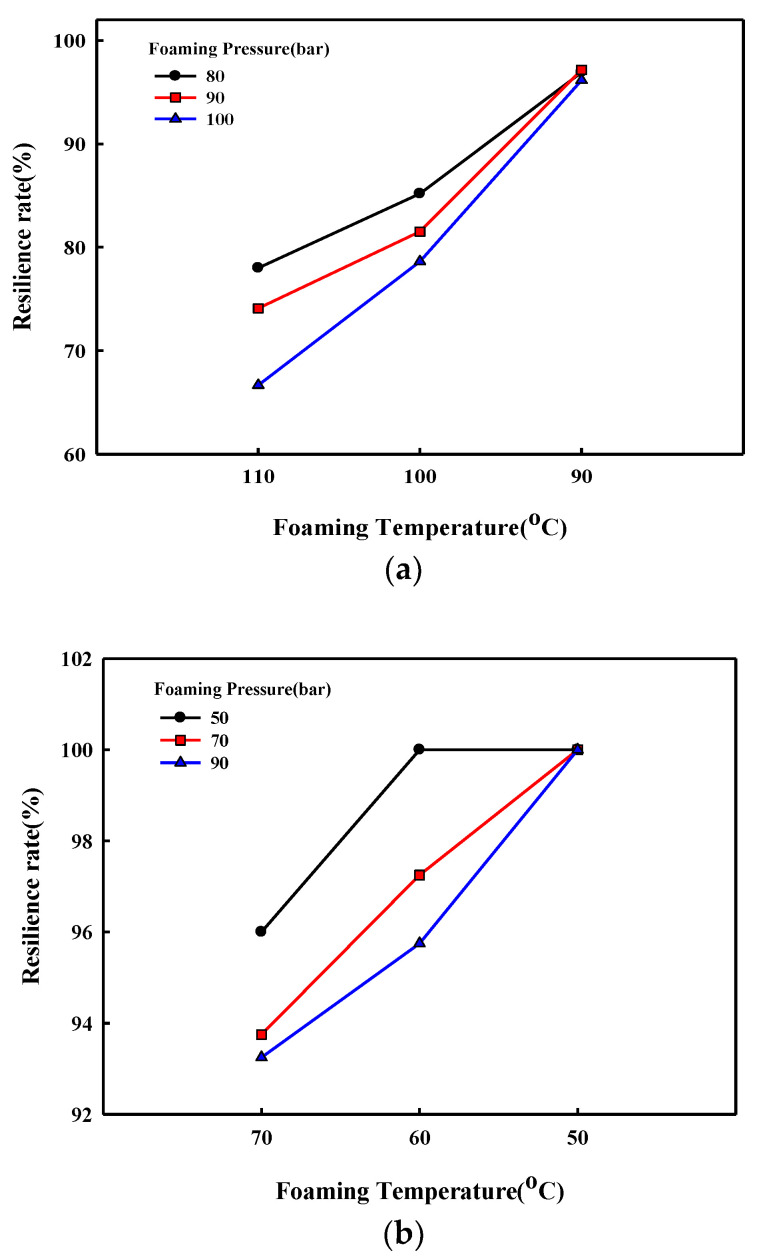
Resilience characteristics of P(3HB-*co*-4HB) foams; (**a**) crystalline P(3HB-*co*-16% 4HB); (**b**) non-crystalline P(3HB-*co*-30% 4HB).

The cell structure of P(3HB-*co*-4HB) foams shown in Figure 3 generally shows thinner cell walls, which hinder the formation of closed cells and result in an open cell structure compared with thermoplastic polyurethane (TPU) foam [39], which is highly elastic, and the cell size is also very irregular. However, the non-crystalline P(3HB-*co*-30% 4HB) foam exhibits thicker walls and more regular size in the closed cell, compared with crystalline P(3HB-*co*-4HB). This can be explained by the crystallinity and their morphology affecting the foaming temperature. In crystalline P(3HB-*co*-4HB), the chain flexibility to foam the cell can be obtained just under the melting temperature, and then the strength for cell nucleation and growth is rapidly lowered with a further increase in temperature, which resulted in uncontrolled cell growth and cell coalescence. However, cell growth and stabilization in non-crystalline P(3HB-*co*-4HB) are more stable because the forming could be archived in a broad range of temperatures with high elastic properties above the glass transition temperature. The cell structure is related to the resiliency against applied pressure. Figure 5 shows the resilience rate of the crystalline P(3HB-*co*-10% 4HB) foam and noncrystalline P(3HB-*co-*30% 4HB) foam. The resilience of P(3HB-*co*-30% 4HB), which is noncrystalline due to the high 4HB content, is greater than that of crystalline P(3HB-*co*-10% 4HB). This is due to the higher elasticity of P(3HB-*co*-30% 4HB) from its higher 4HB content and also due to the thicker cell walls and more regular cell structure. The resilience rate of P(3HB-*co*-30% 4HB) foamed at different temperatures, as shown in Figure 5b and showed much higher resilience for foams prepared at lower temperatures. This shows that the elastic properties of P(3HB-*co*-30% 4HB), along with open/close cell structure, the thickness of the cell walls, and cell size, which in turn depends on an expansion ratio, has a close relationship with resiliency. These results suggest that the possibility of using crystalline P(3HB-*co*-4HB) foams as an environmentally friendly packaging material is higher than that of using it for industrial structural material, which requires elasticity and resiliency to recover to its original form after compression, as it has an open cell structure compared with TPU foams. On the other hand, the non-crystalline P(3HB-*co*-30% 4HB) foams prepared at low temperatures with 100% resilience rates suggest the possibility of developing it as an industrial structural material.

**Figure 6:**
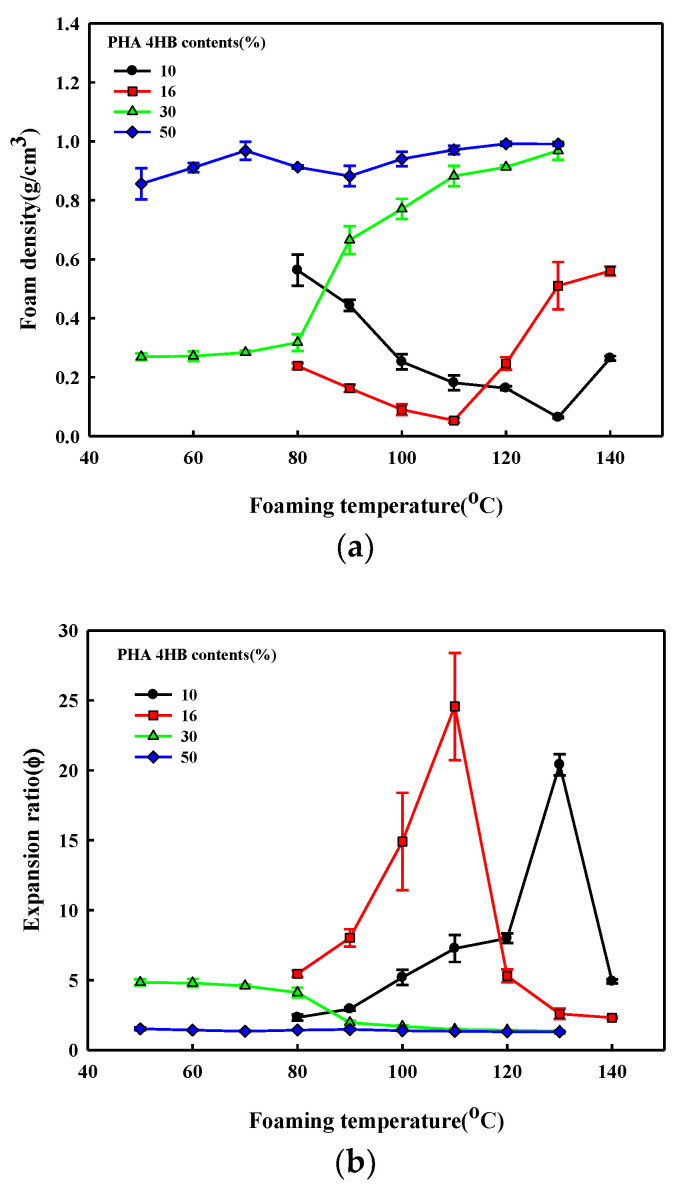
Effect of foaming temperature on the structure of the P(3HB-*co*-4HB) bead foamed at 90 bar; (**a**) foam density; (**b**) expansion ratio.

Figure 6 shows the effect of foaming temperature on the density and the expansion rates calculated from density measurements before and after the expansion of P(3HB-*co*-4HB) beads of different 4HB content. The different foaming characteristic of P(3HB-*co*-30% 4HB) of different 4HB content was confirmed by the SEM micrographs in Figure 3. P(3HB-*co*-50% 4HB) with the highest 4HB content does not show a significant change in density over the range studied, suggesting that cell growth from the diffused supercritical CO_2_ is not possible, due to its high tan δ value. The growth of cells in P(3HB-*co*-30% 4HB) at the foaming temperatures of 50–80 °C due to adequate tan δ and chain mobility can be confirmed. The cell growth of crystalline P(3HB-*co*-10% 4HB) and P(3HB-*co*-16% 4HB) at low temperatures is difficult due to the crystalline structure and lack of chain mobility, but near the melting temperature, chain mobility occurs and the tan δ becomes adequate for foaming to result in cell growth. The highest expansion ratios are obtained at 130 °C and 110 °C for P(3HB-*co*-10% 4HB) and P(3HB-*co*-16% 4HB), respectively. However, the uniformity of cells is very poor, due to the rapid decrease in strength to maintain cells during the stabilization step. The expansion ratio of non-crystalline P(3HB-*co*-30% 4HB) is below 6, while that of crystalline P(3HB-*co*-10% 4HB) and P(3HB-*co*-16% 4HB) is over 20. These P(3HB-*co*-4HB) foams have different expansion ratios, suggesting that they can be used to prepare foams for different applications.

**Figure 7:**
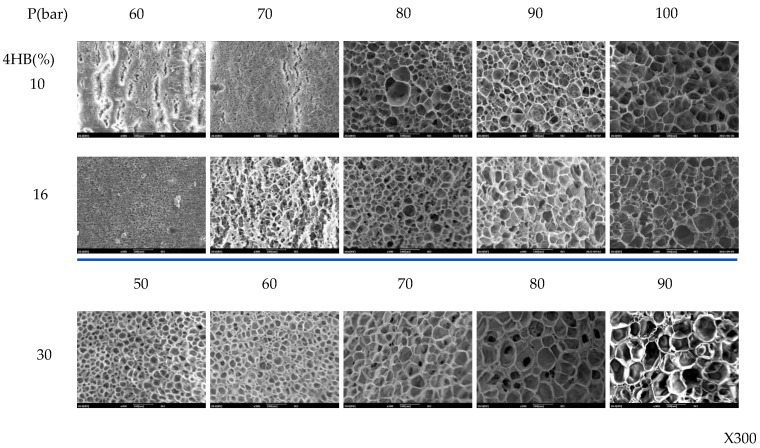
SEM micrographs of P(3HB-*co*-4HB) foamed at various foaming pressure. Foaming temperatures of P(3HB-*co*-10% 4HB), P(3HB-*co*-16% 4HB), and P(3HB-*co*-30% 4HB) are 130 °C, 100 °C, 50 °C, respectively.

**Figure 8:**
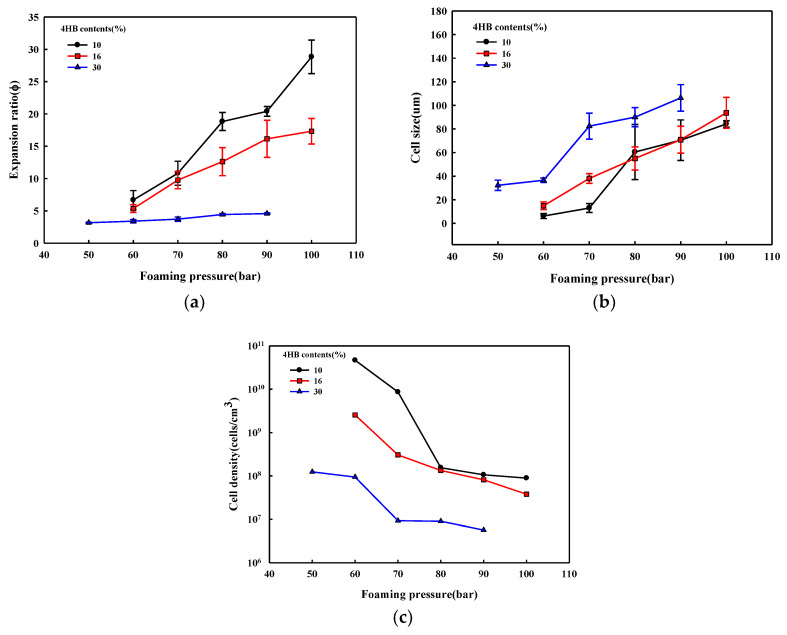
Effect of forming pressure on the structure of the P(3HB-*co*-4HB) foams. Foaming temperatures of P(3HB-*co*-10% 4HB), P(3HB-*co*-16% 4HB), and P(3HB-*co*-30% 4HB) are 130 °C, 100 °C, 50 °C, respectively; (**a**) expansion ratio; (**b**) cell size; (**c**) cell density.

The effect of foaming pressure on foam formation of P(3HB-*co*-4HB) is shown in Figure 7. The foaming temperature was set at the temperature at which the maximum expansion ratio could be obtained from the respective P(3HB-*co*-4HB) at 90 bar. The cell size increases with the increase in pressure. The foam density and expansion ratio of the P(3HB-*co*-4HB) copolymers shown in Figure 8 show a decrease in the foam density and an increase in the expansion ratio with an increase in pressure. An increase in the foaming pressure allows more diffusion of supercritical CO_2_ into P(3HB-*co*-4HB) to form a greater number of nuclei in P(3HB-*co*-4HB) beads, which results in a decrease in the foam density and an increase in the expansion ratio on cell growth when the pressure is relieved. This effect of pressure is more evident in the higher 4HB content P(3HB-*co*-4HB), showing that diffusion of the supercritical CO_2_ is easier in the non-crystalline P(3HB-*co*-4HB) than the crystalline P(3HB-*co*-4HB).

### 3.3. Effect of Chain Extender on Foam Structures

**Figure 9:**
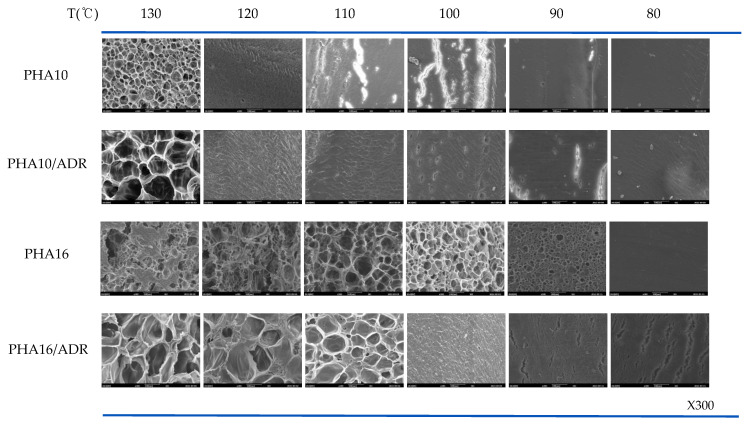
SEM micrographs of foamed crystalline P(3HB-*co*-4HB) with and without chain extender.

**Figure 10:**
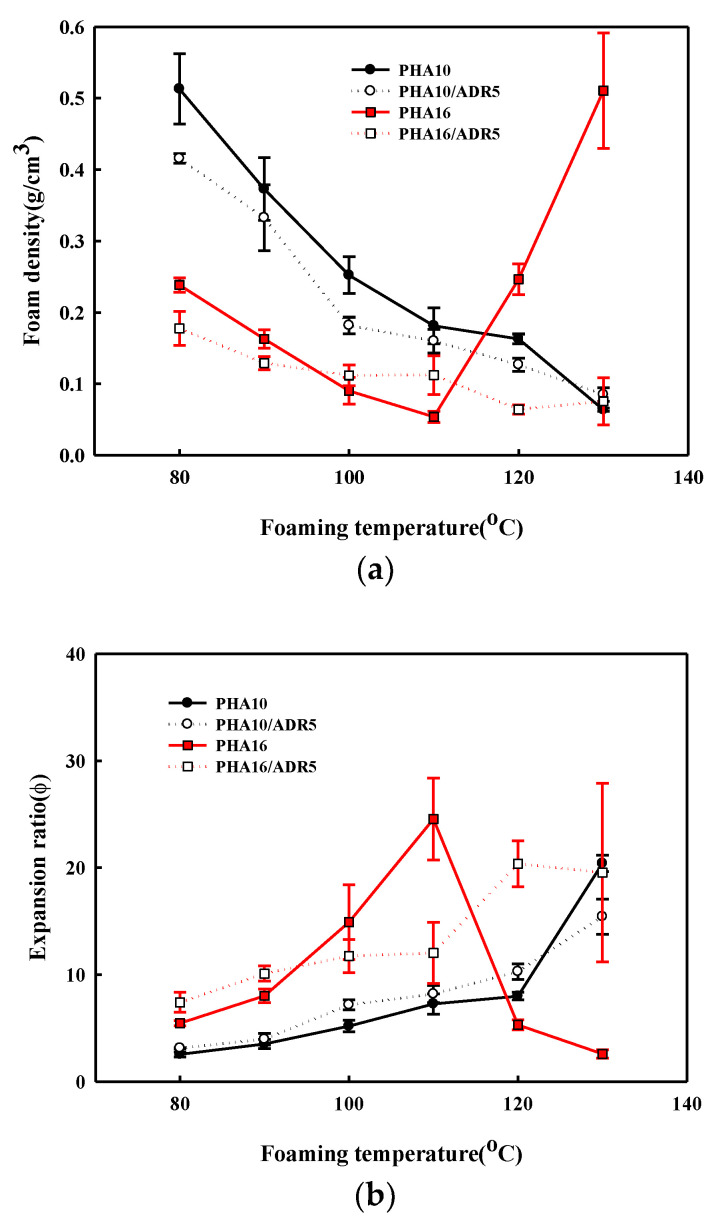
Effect of chain extender on the structure of the P(3HB-*co*-4HB) foams; (**a**) foam density; (**b**) expansion ratio.

SEM micrographs that show the effect of the addition of a chain extender on the supercritical CO_2_ foaming are shown in Figure 9. Although the addition of a chain extender to crystalline P(3HB-*co*-10% 4HB) increases cell size when foamed at 130 °C, it does not affect the temperature window where foaming is possible, while in the case of P(3HB-*co*-16% 4HB), the temperature window appears at a higher temperature and is broadened from 110–100 °C to 120–130 °C. Figure 8b shows that although foaming of non-crystalline P(3HB-*co*-30% 4HB) is possible in the temperature range 50–90 °C in the absence of a chain extender, it is not when a chain extender is added. It was found that the degree of crystallinity of P(3HB-*co*-4HB) modified by a chain extender decreased dramatically, as shown in Table 1. This indicated that the crystalline structural change and the chain flexibility took place by chemical modification. As a result, the tan δ decreases observed in Figure 4 are caused by a structural change in the formation of chain branching or crosslinking from the reaction of epoxy groups of the chain extender Joncryl ADR 4370, containing epoxy groups, and the 3HB hydroxy groups in the crystalline P(3HB-*co*-4HB) of low 4HB content, resulting in a decrease in tan δ. However, in the case of P(3HB-*co*-4HB) of high 4HB content, viscosity decrease occurs instead of the aforementioned increase and decreases tan δ to decrease the foamability. Figure 10 shows the effect of a chain extender on the foam density and expansion ratio of the foam. The addition of the chain extender does not affect the foaming conditions in the case of P(3HB-*co*-10% 4HB) due to relatively high crystallinity, but a general increase in the expansion ratio with a decrease in foam density occurs. However, in the case of P(3HB-*co*-16% 4HB), the change in tan δ with the change in the chain structure allows foaming at temperatures above 110 °C and the foam density remains low above 110 °C to allow foaming at 120–130 °C, where foaming was not possible in the absence of a chain extender and an expansion ratio of around 20 can be obtained. Therefore, the control of the foaming properties with a chain extender is only possible in crystalline P(3HB-*co*-16% 4HB) with reasonably low crystallinity and tan δ value.

## 4. Conclusions

Biodegradable P(3HB-*co*-4HB) of different 4HB contents and foam beads processed with supercritical carbon dioxide was used to study the effects of 4HB content on the foaming conditions and foam properties. It was found that various foam structures for packaging and industrial materials were obtained by controlling the 4HB content in P(3HB-*co*-4HB), which resulted in changes in the crystallinity and viscoelastic properties of P(3HB-*co*-4HB). The low 4HB content P(3HB-*co*-4HB) that maintained a crystalline structure can be foamed just under the melting temperature, but the range of the foaming temperature becomes broader with an increase in the 4HB content. The expansion ratio is around 20 and an open cell structure is formed. The non-crystalline P(3HB-*co*-4HB) of high 4HB content has a relatively wide window of foaming around the temperature at which chain mobility occurs. The formed foam has thicker cell walls, expansion ratios of about 6, and the cell size is regular, resulting in enhanced resiliency against compression, compared with crystalline P(3HB-*co*-4HB). The chain extender changes the chain structure of crystalline P(3HB-*co*-16% 4HB), which changes the crystallinity and chain mobility and, thus, changes the temperature at which foaming is possible and the cell structure. To enhance the physical properties of P(3HB-*co*-4HB) foams, future studies for the blends and composites using P(3HB-*co*-4HB) may be considered.

## Figures and Tables

**Table 1:** Characteristics of P(3HB-co-4HB) used in this study.

Sample Code [Polymer]	4HB Content (%)	M_w_ (k)	$\Delta H_{m}\;(J/g)$	T_m1_(°C)	T_m2_(°C)	T_c_(°C)
PHA10 [P(3HB-*co*-10% 4HB)]	10	600	23.97	129.96	145.92	75.82
PHA10 with ADR(5phr)	10	600	9.65	113.59	141.96	72.53
PHA10 [P(3HB-*co*-16% 4HB)]	16	1000	3.99	115.82	155.59	59.67
PHA10 with ADR(5phr)	16	1000	2.54	113.55	154.96	56.84
PHA10 [P(3HB-*co*-30% 4HB)]	30	687	-	-	-	-
PHA10 [P(3HB-*co*-50% 4HB)]	53.7	901	-	-	-	-

## Data Availability

Data are in the authors’ possession.
